# Dotinurad: a clinical pharmacokinetic study of a novel, selective urate reabsorption inhibitor in subjects with hepatic impairment

**DOI:** 10.1007/s10157-019-01816-4

**Published:** 2019-11-23

**Authors:** Yuji Kumagai, Masashi Sakaki, Kenichi Furihata, Takayoshi Ito, Kazuaki Inoue, Takafumi Yoshida, Shigeki Matsumoto, Kazuki Furuno, Atsushi Hagino

**Affiliations:** 1 Kitasato University School of Medicine, 1-15-1, Kitasato, Minami-ku, Sagamihara, Kanagawa 252-0374 Japan; 2 Showa University Hospital, 1-5-8, Hatanodai, Shinagawa-ku, Tokyo, 142-8666 Japan; 3 P-One Clinic, Keikokai Medical Corporation, 8-1 Yokamachi, Hachioji, Tokyo 192-0071 Japan; 4 Digestive Disease Center, Showa University Koto Toyosu Hospital, 5-1-38, Toyosu, Koto-ku, Tokyo, 135-8577 Japan; 5 Showa University Fujigaoka Hospital, 1-30, Fujigaoka, Aoba-ku, Yokohama, Kanagawa 227-8501 Japan; 6 Kurume Clinical Pharmacology Clinic, 67, Asahimachi, Kurume, Fukuoka 830-0011 Japan; 7 Clinical Research Department, Mochida Pharmaceutical Co., Ltd, 1-22 Yotsuya, Shinjuku-ku, Tokyo, 160-0004 Japan

**Keywords:** Dotinurad, Pharmacokinetics, Pharmacodynamics, URAT1 inhibitor, Selective urate reabsorption inhibitor (SURI), Hepatic impairment

## Abstract

**Background:**

Dotinurad is a novel, selective urate reabsorption inhibitor, which reduces serum uric acid levels by inhibiting the urate transporter 1 (URAT1). We compared the pharmacokinetics (PK), pharmacodynamics (PD), and safety of dotinurad in subjects with hepatic impairment and normal hepatic function.

**Methods:**

This was a multicenter, open-label, single dose study. A total of 24 subjects were divided into four groups: the normal hepatic function group and the mild, moderate, and severe hepatic impairment groups. The primary endpoints were changes in plasma dotinurad levels and PK parameters.

**Results:**

The geometric mean ratio of the maximum plasma concentration (*C*_max_) [two-sided 90% confidence interval (CI)] of dotinurad in in the mild, moderate, and severe hepatic impairment groups relative to that in the normal hepatic function group was 0.840 (0.674–1.047), 0.798 (0.653–0.976), and 0.747 (0.570–0.979), respectively, showing a lower *C*_max_ in the moderate and severe hepatic impairment groups. Following adjustment for body weight, only the moderate hepatic impairment group had a lower *C*_max_ than the normal hepatic function group. No meaningful differences in other PK parameters were observed between the groups. Regarding the PD of dotinurad, the changes in serum uric acid levels after dosing were similar in all groups. As for safety, no noteworthy concerns were raised in relation to any group.

**Conclusion:**

The study revealed no clinically meaningful influence of hepatic impairment on the PK, PD, or safety of dotinurad. These findings indicate possibility that dotinurad can be used without dose adjustment in patients with hepatic impairment.

**Electronic supplementary material:**

The online version of this article (10.1007/s10157-019-01816-4) contains supplementary material, which is available to authorized users.

## Introduction

With the westernization of dietary habits, hyperuricemia with or without gout are becoming increasingly prevalent and even spreading to younger people in Japan. The prevalence of hyperuricemia in men in their 30s has reached 30% [1] and that of gout in men exceeds 1% [2]. Against this background, gout and hyperuricemia are now being categorized as lifestyle-related diseases along with hypertension, dyslipidemia, and diabetes mellitus [3]. Hyperuricemia can cause urate deposition diseases including gouty arthritis [3] and appropriate control of uric acid levels is critical for the prevention of such disorders.

The Japanese guideline recommends the treatment of hyperuricemia using xanthine oxidoreductase inhibitors (XOIs) for the overproduction type and uricosuric drugs for the underexcretion type. In Japan, there is a long history of use of the uricosuric drug benzbromarone and the XOI allopurinol [4]. Since 2000, XOI febuxostat and topiroxostat have been developed, thus increasing treatment options in recent years.

However, these existing drugs have safety issues such as liver toxicity. Benzbromarone, the most common uricosuric drug in Japan, is reported to cause serious liver disorder as an adverse drug reaction (ADR) [5]. Therefore this drug is contraindicated in patients with hepatic disorders in Japan and has been withdrawn in the US and some EU countries. XOIs (allopurinol, febuxostat, and topiroxostat) are also reported to cause liver disorder as a severe ADR on rare occasions [6–8] and should be administered carefully in patients with hepatic disorders. Therefore, development of antihyperuricemics that can be used in patients with hepatic impairment is expected to add new options in the treatment of hyperuricemia.

Dotinurad is a novel, selective urate reabsorption inhibitor (SURI), which reduces serum uric acid levels via selective inhibition of urate transporter 1 (URAT1). URAT1 are expressed on the proximal renal tubules and is responsible for reabsorption of uric acid [9].

Completed phase 2 studies of dotinurad demonstrated a potent uric acid lowering effect as well as a favorable safety profile [10, 11]. In a phase 1 study in healthy adults, PK parameters of dotinurad showed a linear *C*_max_ and its time to *C*_max_ (*T*_max_) and elimination half-life (*T*_1/2_) were approximately 3 and 10 h, respectively, which were nearly constant irrespective of the dose level. The results of a non-clinical study and a clinical pharmacology study in healthy adults demonstrated that glucuronate and sulfate conjugates, the primary metabolites of dotinurad, were mostly excreted in urine. Based on the evidence that elimination of dotinurad from plasma depends primarily on hepatic clearance, we conducted this study to compare the PK, PD, and safety between subjects with hepatic impairment and those with normal hepatic function.

## Methods

### Study design

This was a multicenter, open-label, single-dose, clinical pharmacology study conducted at five clinical institutions in Japan.

In this study, the target number of subjects was determined to be a total of 24 subjects, six per group: the mild hepatic impairment group, the moderate hepatic impairment group, the severe hepatic impairment group, and the normal hepatic function group. The severity of hepatic impairment in subjects with cirrhosis was determined using the Child–Pugh score for severity-based grouping of subjects. Subjects with a Child–Pugh score of 5–6 (class A), 7–9 (class B), and 10–15 (class C) points were respectively included in the mild, moderate, and severe hepatic impairment groups. The normal hepatic function group included subjects whose age and body weight were similar to means of those in subjects with mild and moderate hepatic impairment.

### Inclusion and exclusion criteria

The common inclusion criteria for all subjects were as follows: age 20 years and older; and BMI ≥ 18.5 kg/m^2^ to < 30.0 kg/m^2^ at screening. Inclusion criteria to subjects with hepatic impairment were as follows: a diagnosis of hepatic cirrhosis and Child–Pugh Class any of A, B, or C at screening. Inclusion criteria to subjects with normal hepatic function were as follows: ALT (GPT) and AST (GOT) at screening < 1.25 times the upper limit of normal and normal hepatic function in the opinion of the investigator; the age at the time of consent was within the mean age ± 10 years of the mild and moderate hepatic impairment group subjects; and the body weight at screening was within the mean body weight ± 20% of the mild and moderate hepatic impairment group subjects.

The common exclusion criteria for all subjects were as follows: subjects with, or with a history of, any cardiac, renal, pulmonary, hematological, gastrointestinal, thyroidal, neuropsychiatric, metabolic/electrolyte disorders that would make their participation in the study unsuitable in the opinion of the investigator; subjects with a history of liver transplantation; subjects who donated a blood component within two weeks of study administration; subjects who donated ≥ 200 mL of whole blood within four weeks of study administration or ≥ 400 mL of whole blood within 12 weeks (men) or 16 weeks (women) of study administration; individuals who donated ≥ 1000 mL (men) or ≥ 600 mL (women) of whole blood within 52 weeks of study administration; subjects who had renal calculi or clinical symptoms of urinary calculi (e.g., hematuria and back pain) at screening; and subjects with estimated glomerular filtration rate (eGFR) ˂ 45 mL/min/1.73 m^2^. Exclusion criteria to subjects with hepatic impairment were as follows: subjects who were unlikely to abstain from drinking during hospitalization in the opinion of the investigator; subjects with ascites requiring invasive treatment; and subjects with ≥ grade 2 hepatic encephalopathy according to the Japan Coma Scale at screening. Exclusion criteria to subjects with normal hepatic function were as follows: subjects with symptoms of alcoholism or with a history of alcoholism; and subjects with a positive test for HBs antigen or HCV antibody at screening.

### Study schedule and measurements

Subjects with hepatic impairment underwent eligibility assessment including Child–Pugh classification at screening. Eligible subjects were admitted to the study site one or two days before the observation period. On the first day of the observation period, they received a single oral dose of dotinurad at 4 mg, the estimated maximum clinical dose, following at least 10 h of fasting. They remained hospitalized for another 48 h or longer, during which the scheduled observations/examinations and assessments were conducted. After safety was confirmed, they were discharged and then returned to the study site between six and 10 days postdose for follow-up study.

Blood samples for determination of plasma dotinurad levels were collected at 12 time points: baseline, 0.5, 1, 2, 3, 4, 6, 8, 12, 24, 36, and 48 h postdose. Blood samples for calculation of the plasma protein binding rate of dotinurad were collected at baseline. Urine samples for determination of urinary metabolites levels were collected on the day before study administration, 0–6, 6–12, 12–24, and 24–48 h postdose.

For PD assessment, blood samples for determination of serum uric acid levels were collected at 10 time points: baseline, 1, 2, 4, 6, 8, 12, 24, 36, and 48 h postdose. Urine samples collected for PK assessment were also used for determination of urinary uric acid levels.

During the study, ingestion of foods and drinks other than prescribed ones and alcohol consumption were prohibited.

### Pharmacokinetic analysis methods

#### Measurement method

Plasma levels of dotinurad were determined by liquid chromatography tandem mass spectrometry (LC–MS/MS) method (LC: LC-20AD system, SHIMADZU. MS/MS: API4000, SCIEX) at Sekisui Medical Co., Ltd. (Tokyo, Japan). The lower limit of quantification (LLOQ) was 1 ng/mL.

Urinary levels of metabolites (glucuronate conjugate and sulfate conjugate) were determined by LC–MS/MS method (LC: Nexera X2, Prominence, SHIMADZU. MS/MS: Triple Quad 4500, SCIEX) at Fuji Yakuhin Co., Ltd. (Saitama, Japan). The LLOQ was 10 ng/mL.

In order to calculate the unbound fraction in plasma, dotinurad was added to baseline plasma samples, which were then ultrafiltered and underwent LC–MS/MS measurement (LC: LC-20AD system, SHIMADZU. MS/MS: API4000, SCIEX) at Sekisui Medical Co., Ltd. The LLOQ was 0.1 ng/mL.

### Statistical analyses

#### Pharmacokinetic parameters

The summary statistics of plasma dotinurad levels at individual time points were calculated for each group and their changes over time were plotted. The summary statistics of PK parameters were also calculated for each group. Phoenix^®^ WinNonlin^®^ (Ver. 6.1, Certara L.P., Princeton, NJ, USA) was used in noncompartmental analysis to determine the following PK parameters: *C*_max_, *T*_max_, *T*_1/2_, area under the plasma concentration–time curve from time 0 to 48 h (AUC_0–48_) or from time 0 to infinity (AUC_0–inf_), total clearance/fraction of dose absorbed (CL_tot_/*F*), and distribution volume/fraction of dose absorbed (Vd/*F*). We calculated the geometric mean ratio of the PK parameters determined in each hepatic impairment group, relative to those in the normal hepatic function group, together with their two-sided 90% CIs.

The summary statistics of the amount excreted from time 0 to 48 h (Ae_0–48_) and the fraction of the dose excreted in urine (fe) as urinary metabolites were calculated for each group. Likewise for dotinurad, the summary statistics of the bound fraction in plasma and the unbound fraction in plasma were calculated for each group.

#### Pharmacodynamic parameters

The summary statistics of serum uric acid levels at individual time points were calculated for each group, and their changes over time were plotted. The summary statistics of urinary uric acid excretion in each 24-h interval were calculated for each group, and their changes over time were plotted. In addition, the summary statistics of PD parameters [delta maximum effective concentration (∆EC_max_), delta area under the serum uric acid concentration–time curve (∆AUEC) from time 0 to 48 h (∆AUEC_0–48_), Ae_0–48_, renal clearance of uric acid (CL_UR_), and fractional excretion of uric acid (FEUA)] of uric acid were calculated for each group. We calculated the mean difference in determined PD parameters in each hepatic impairment group relative to those in the normal hepatic function group, together with their two-sided 90% CIs.

#### Safety analyses

The investigator observed/examined/assessed AEs, subjective symptoms and objective findings, vital signs, body weight, electrocardiography, and laboratory test values. AEs were coded by preferred term using MedDRA (version 21.0; Japanese Maintenance Organization, Tokyo, Japan) and were tabulated by group.

## Results

### Baseline characteristics

A total of 24 subjects (six in the normal hepatic function group, six in the mild hepatic impairment group, nine in the moderate hepatic impairment group, and three in the severe hepatic impairment group) received dotinurad. No subjects discontinued the study after study administration. All administered subjects were included in the PK, PD and safety analyses.

Age in administered subjects was similar among the four groups. Body weight [mean ± standard deviation (SD)] was 61.23 ± 5.71 kg in the normal hepatic function group, 66.47 ± 9.43 kg in the mild hepatic impairment group, 61.67 ± 12.81 kg in the moderate hepatic impairment group, and 72.27 ± 10.13 kg in the severe hepatic impairment group. The Child–Pugh score in the mild, moderate, and severe hepatic impairment groups was 5.2 ± 0.4, 7.8 ± 0.8, and 10.0 ± 0.0 points, respectively (Table 1).

**Table 1:** Baseline characteristics

Characteristic	Group				Overall (*n* = 24)
	Normal hepatic function (*n* = 6)	Mild hepatic impairment (*n* = 6)	Moderate hepatic impairment (*n* = 9)	Severe hepatic impairment (*n* = 3)	
Age (years)					
Mean ± SD	57.8 ± 6.8	64.2 ± 8.4	56.2 ± 10.2	63.7 ± 4.0	59.5 ± 8.7
Min–Max	50–66	48–72	40–70	59–66	40–72
Height (cm)					
Mean ± SD	167.08 ± 6.66	160.53 ± 14.60	163.99 ± 8.97	161.87 ± 4.82	163.63 ± 9.59
Min–Max	156.9–176.2	134.3–176.0	149.0–176.1	156.4–165.5	134.3–176.2
Body weight (kg)					
Mean ± SD	61.23 ± 5.71	66.47 ± 9.43	61.67 ± 12.81	72.27 ± 10.13	64.08 ± 10.34
Min–Max	52.5–68.9	49.5–76.3	45.1–82.1	62.6–82.8	45.1–82.8
BMI (kg/m^2^)					
Mean ± SD	22.01 ± 2.61	25.96 ± 3.63	22.74 ± 3.01	27.59 ± 3.70	23.97 ± 3.61
Min–Max	19.2–26.4	20.7–30.2	20.1–28.4	23.4–30.2	19.2–30.2
Sex					
* n* (%)					
Male	5 (83.3)	4 (66.7)	7 (77.8)	2 (66.7)	18 (75.0)
Female	1 (16.7)	2 (33.3)	2 (22.2)	1 (33.3)	6 (25.0)
Race					
* n* (%)					
Asian	6 (100.0)	6 (100.0)	9 (100.0)	3 (100.0)	24 (100.0)
Other	0 (0.0)	0 (0.0)	0 (0.0)	0 (0.0)	0 (0.0)
Child–Pugh score					
Mean ± SD	–	5.2 ± 0.4	7.8 ± 0.8	10.0 ± 0.00	7.3 ± 1.8
Min–Max	–	5–6	7–9	10–10	5–10
eGFR^a^					
Mean ± SD	74.7 ± 7.6	68.3 ± 13.0	83.8 ± 36.3	88.7 ± 17.5	78.3 ± 24.3
(mL/min/1.73m^2^)					
Min–Max	63–86	50–86	47–156	74–108	47–156
Ccr					
Mean ± SD	124.8 ± 20.0	112.0 ± 38.1	109.3 ± 47.2	112.7 ± 26.3	114.3 ± 35.8
(mL/min/1.73m^2^)					
Min–Max	98–147	65–159	72–195	96–143	65–195
Current disease^b^					
* n* (%)					
No	6 (100.0)	1 (16.7)	0 (0.0)	0 (0.0)	7 (29.2)
Yes	0 (0.0)	5 (83.3)	9 (100.0)	3 (100.0)	17 (70.8)
Smoking habit					
*n* (%)					
No	4 ( 66.7)	3 ( 50.0)	3 ( 33.3)	1 ( 33.3)	11 ( 45.8)
Yes	2 ( 33.3)	3 ( 50.0)	6 ( 66.7)	2 ( 66.7)	13 ( 54.2)

^a^eGFR for male (mL/min/1.73m^2^) = 194 × Serum creatinine^−1.094^ × age^−0.287^

eGFR for female (mL/min/1.73m^2^) = 194 × Serum creatinine^−1.094^ × age^−0.287^ × 0.739

^b^Other than hepatic cirrhosis

### Pharmacokinetics

**Fig. 1:**
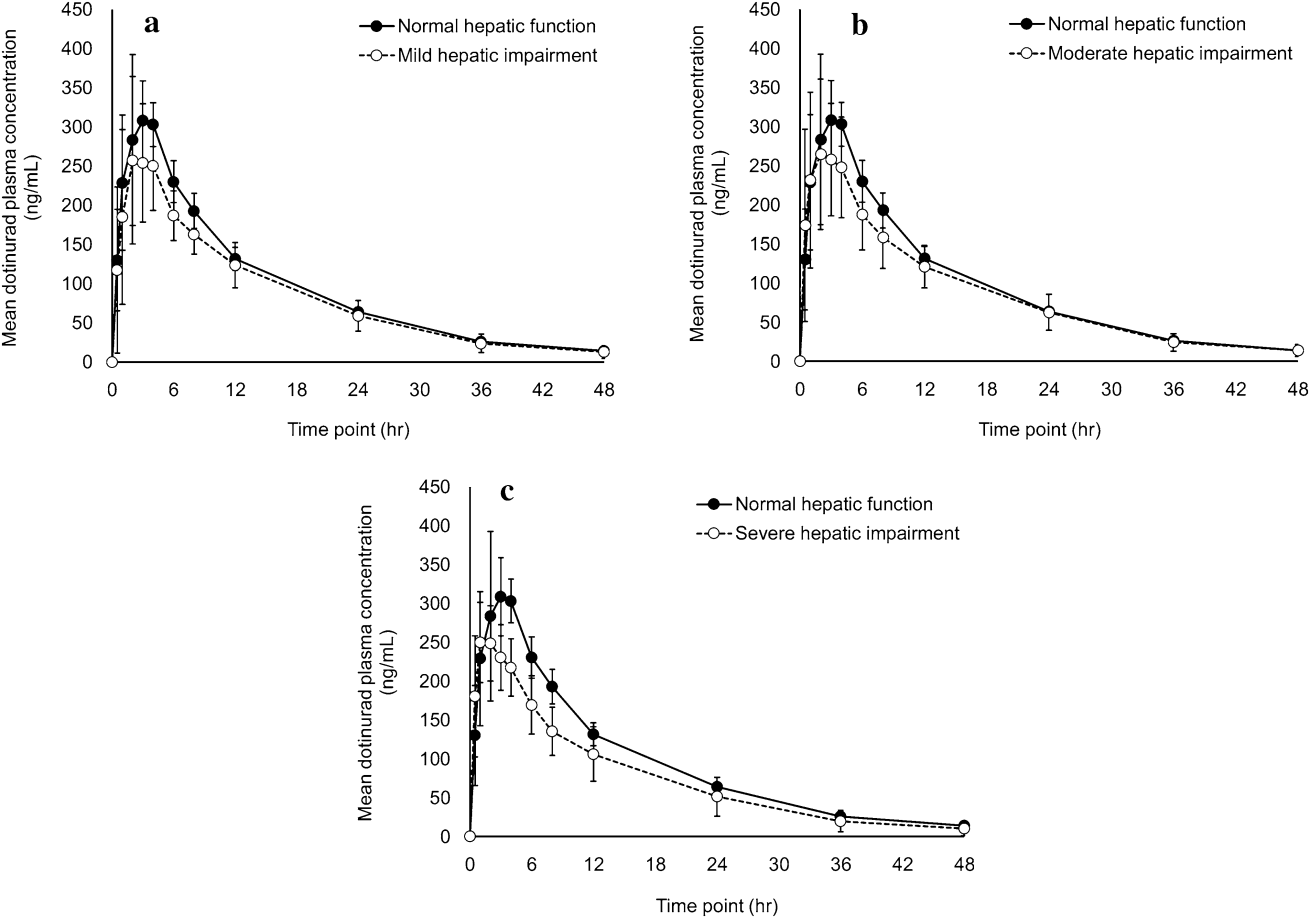
Mean (± SD) plasma concentration versus time profiles for dotinurad. **a** Normal hepatic function and mild hepatic impairment group. *SD* standard deviation. **b** Normal hepatic function and moderate hepatic impairment group. *SD* standard deviation. **c **Normal hepatic function and severe hepatic impairment group. *SD* standard deviation

The mean plasma dotinurad level followed a similar time course in all groups, peaking at 1–3 h postdose (Fig. 1, Supplement 1), with the peak level being lower in the hepatic impairment groups than in the normal hepatic function group. Adjustment for body weight reduced the difference in the peak level between the normal hepatic function group and the mild and severe hepatic impairment groups (Supplement 2). The mean plasma dotinurad level in the elimination phase followed a similar time course in all groups.

*C*_max_ [point estimate (two-sided 90% CI)] in the moderate and severe hepatic impairment groups [0.798 (0.653–0.976) and 0.747 (0.570–0.979), respectively] was the only parameter for which the upper limit of two-sided 90% CI of the geometric mean ratio relative to the normal hepatic function group was less than 1 (Table 2). Following adjustment for body weight, the upper limit of the 90% CI for *C*_max_ was less than 1 in the moderate hepatic impairment group (Table 3).

**Table 2:** Pharmacokinetic parameters of dotinurad in plasma, with comparison between groups

Parameters (unit)	Normal hepatic function (*n* = 6)	Mild hepatic impairment (*n* = 6)		
	Mean ± SD	Mean ± SD	Geometric mean ratio^a^	
			Point estimate	Two-sided 90% CI
*C*_max_ (ng/mL)	339.15 ± 28.57	289.88 ± 65.03	0.840	0.674–1.047
*T*_max_ (h)	2.67 ± 1.03	2.17 ± 1.17	–	–
*T*_1/2_ (h)	10.80 ± 0.55	10.50 ± 2.42	0.953	0.792–1.147
AUC_0–48_ (ng h/mL)	4541.72 ± 343.39	4013.66 ± 823.02	0.869	0.693–1.091
AUC_0–inf_ (ng h/mL)	4761.81 ± 369.35	4234.01 ± 950.16	0.872	0.684–1.112
CL_tot_/*F* (L/h)	0.844 ± 0.066	0.989 ± 0.240	1.147	0.900–1.463
Vd/*F* (L)	13.16 ± 1.19	14.52 ± 2.36	1.094	0.905–1.322

Parameters (unit)	Moderate hepatic impairment (*n* = 9)			Severe hepatic impairment (*n* = 3)		
	Mean ± SD	Geometric mean ratio^a^		Mean ± SD	Geometric mean ratio^a^	
		Point estimate	Two-sided 90% CI		Point estimate	Two-sided 90% CI
*C*_max_ (ng/mL)	280.34 ± 87.91	0.798	0.653–0.976	255.23 ± 46.06	0.747	0.570–0.979
*T*_max_ (h)	2.44 ± 1.01	–	–	1.33 ± 0.58	–	–
*T*_1/2_ (h)	10.75 ± 2.28	0.978	0.826–1.158	9.82 ± 2.47	0.892	0.711–1.119
AUC_0–48_ (ng h/mL)	4095.91 ± 1133.49	0.875	0.711–1.077	3592.84 ± 1173.52	0.765	0.579–1.011
AUC_0–inf_ (ng h/mL)	4327.09 ± 1249.48	0.879	0.704–1.098	3757.37 ± 1343.74	0.758	0.563–1.021
CL_tot_/*F* (L/h)	0.991 ± 0.262	1.137	0.911–1.420	1.159 ± 0.404	1.319	0.980–1.777
Vd/*F* (L)	14.99 ± 3.69	1.112	0.935–1.323	15.51 ± 1.97	1.177	0.933–1.485

*AUC*_*0–inf*_ area under the plasma concentration − time curve from time 0 to infinity, *AUC*_*0–48*_ area under the plasma concentration − time curve from time 0 to 48 h, *CI* confidence interval, *CL*_*tot*_*/F* total clearance/fraction of dose absorbed, *C*_*max*_ maximum plasma concentration, *SD* standard deviation, *T*_*max*_ time to maximum plasma concentration, *T*_*1/2*_ elimination half-life, *Vd/F* distribution volume/fraction of dose absorbed

^a^The mean was converted to a common logarithm and then the geometric mean ratio was calculated using the following formula:

Geometric mean ratio = 10^Mean difference^

Mean difference = (mean in the target group) − (mean in the normal hepatic function group)

**Table 3:** Pharmacokinetic parameters (adjusted for body weight) of dotinurad in plasma, with comparison between groups

Parameters (unit)	Normal hepatic function (*n* = 6)	Mild hepatic impairment (*n* = 6)		
	Mean ± SD	Mean ± SD	Geometric mean ratio^a^	
			Point estimate	Two-sided 90% CI
*C*_max_ (ng kg/mL)	20,665.9 ± 1450.3	19,054.3 ± 3867.7	0.908	0.720–1.147
AUC_0–48_ (ng kg h/mL)	277,096.0 ± 22,379.2	265,029.3 ± 58,564.3	0.940	0.710–1.245
AUC_0–inf_ (ng kg h/mL)	290,502.3 ± 23,625.3	279,743.3 ± 67,960.8	0.943	0.697–1.275
CL_tot_/*F* (L/h/kg)	0.0139 ± 0.0012	0.0150 ± 0.0035	1.061	0.784–1.435
Vd/*F* (L/kg)	0.216 ± 0.021	0.220 ± 0.032	1.011	0.834–1.226

Parameters (unit)	Moderate hepatic impairment (*n* = 9)			Severe hepatic impairment (*n* = 3)		
	Mean ± SD	Geometric mean ratio^a^		Mean ± SD	Geometric mean ratio^a^	
		Point estimate	Two-sided 90% CI		Point estimate	Two-sided 90% CI
*C*_max_ (ng kg/mL)	16,994.2 ± 4765.3	0.792	0.640–0.980	18,181.2 ± 3757.9	0.869	0.653–1.155
AUC_0–48_ (ng kg h/mL)	252,948.5 ± 84,717.2	0.868	0.672–1.122	258,929.9 ± 94,795.0	0.889	0.630–1.255
AUC_0–inf_ (ng kg h/mL)	268,605.8 ± 97,755.3	0.872	0.662–1.149	270,778.5 ± 105,677.4	0.881	0.609–1.275
CL_tot_/*F* (L/h/kg)	0.0169 ± 0.0065	1.146	0.870–1.510	0.0167 ± 0.0077	1.135	0.784–1.642
Vd/*F* (L/kg)	0.247 ± 0.062	1.121	0.940–1.337	0.222 ± 0.056	1.012	0.800–1.282

*AUC*_*0–inf*_ area under the plasma concentration−time curve from time 0 to infinity, *AUC*_*0–48*_ area under the plasma concentration−time curve from time 0 to 48 h, *CI* confidence interval, *CL*_*tot*_*/F* total clearance/fraction of dose absorbed, *C*_*max*_ maximum plasma concentration, *SD* standard deviation, *Vd/F* distribution volume/fraction of dose absorbed

^a^The mean was converted to a common logarithm and then the geometric mean ratio was calculated using the following formula:

Geometric mean ratio = 10^Mean difference^

Mean difference = (mean in the target group) − (mean in the normal hepatic function group)

**Fig. 2:**
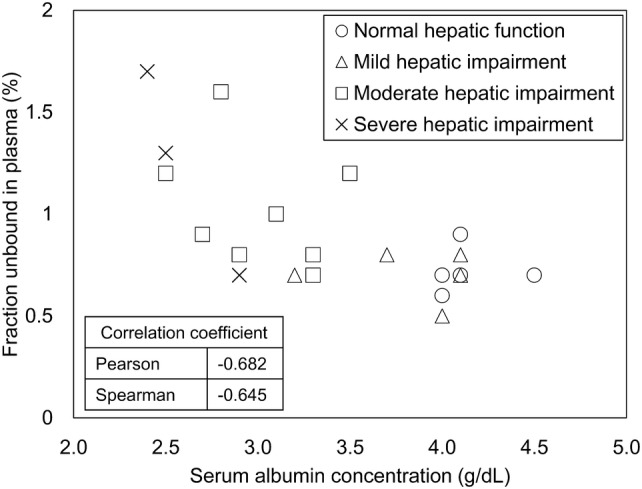
Correlation between unbound fraction in plasma and serum albumin levels

The unbound fraction in plasma of dotinurad was higher in the moderate and severe hepatic impairment groups than in the normal hepatic function group (Table 4) and had a negative correlation with serum albumin levels (Fig. 2).

**Table 4:** Summary statistics of bound fraction rate and unbound fraction rate in plasma

	Normal hepatic function (*n* = 6)	Mild hepatic impairment (*n* = 6)	Moderate hepatic impairment (*n* = 9)	Severe hepatic impairment (*n* = 3)
	Mean ± SD	Mean ± SD	Mean ± SD	Mean ± SD
Bound fraction rate in plasma (%)	99.28 ± 0.10	99.30 ± 0.11	98.98 ± 0.28	98.77 ± 0.50
Unbound fraction rate in plasma (%)	0.72 ± 0.10	0.70 ± 0.11	1.02 ± 0.28	1.23 ± 0.50

*SD* standard deviation

The fe of the glucuronate conjugate was similar in all groups. The cumulative urinary excretion rate of the sulfate conjugate was also similar in all groups, but lower in the moderate hepatic impairment group than in the normal hepatic function group (Table 5).

**Table 5:** Summary statistics of pharmacokinetic parameters of urinary dotinurad metabolites

	Urinary dotinurad metabolites	PK parameter	Mean ± SD
Normal hepatic function (*n* = 6)	Glucuronate conjugate	Ae_0–*t*_ (μg)	2216.42 ± 292.39
		fe (%)	37.1463 ± 4.9004
	Sulfate conjugate	Ae_0–*t*_ (μg)	830.24 ± 197.17
		fe (%)	16.9644 ± 4.0287
Mild hepatic impairment (*n* = 6)	Glucuronate conjugate	Ae_0–*t*_ (μg)	2143.31 ± 560.03
		fe (%)	35.9210 ± 9.3859
	Sulfate conjugate	Ae_0–*t*_ (μg)	774.71 ± 290.29
		fe (%)	15.8297 ± 5.9316
Moderate hepatic impairment (*n* = 9)	Glucuronate conjugate	Ae_0–*t*_ (μg)	2160.27 ± 404.02
		fe (%)	36.2053 ± 6.7712
	Sulfate conjugate	Ae_0–*t*_ (μg)	397.60 ± 242.86
		fe (%)	8.1242 ± 4.9623
Severe hepatic impairment (*n* = 3)	Glucuronate conjugate	Ae_0–*t*_ (μg)	2257.53 ± 418.86
		fe (%)	37.8354 ± 7.0199
	Sulfate conjugate	Ae_0–*t*_ (μg)	741.60 ± 481.02
		fe (%)	15.1531 ± 9.8288

*Ae*_*0–t*_ amount of drug excreted in urine from time 0 to 48 h, *fe* fraction of dose excretion in urine from time 0 to 48 h, *SD* standard deviation

### Pharmacodynamics

**Fig. 3:**
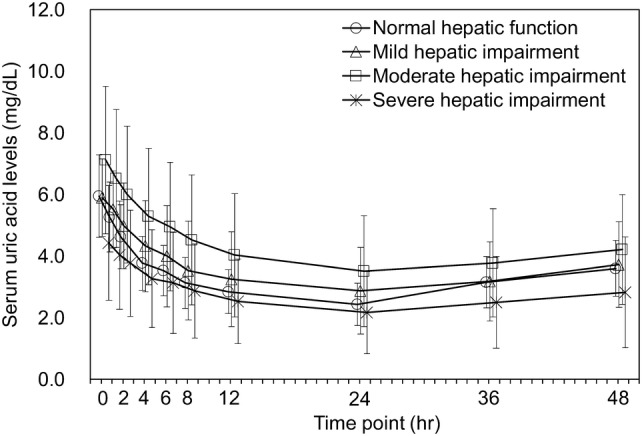
Mean (± SD) serum uric acid concentration versus time profiles. *SD* standard deviation

**Fig. 4:**
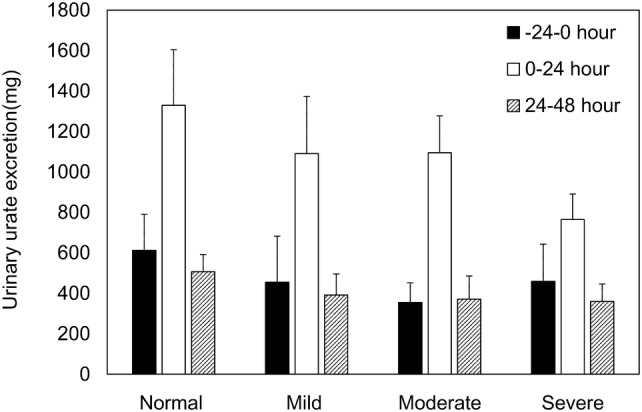
Mean (± SD) urinary uric acid excretion versus time profiles. *SD* standard deviation

The baseline serum uric acid level (mean ± SD) in the normal hepatic function group and the mild, moderate, and severe hepatic impairment groups was 5.95 ± 1.34 mg/dL, 5.90 ± 1.26 mg/dL, 7.12 ± 2.39 mg/dL, and 4.43 ± 1.86 mg/dL, respectively. The mean serum uric acid level was lowest at 24 h postdose in all groups, followed by an elevation, and remained lower than the baseline even at 48 h postdose (Fig. 3). The urinary uric acid excretion from time zero to 24 h (Ae_0–24_) was greater than the baseline (Ae_−24–0_) and that from time 24 to 48 h (Ae_24–48_) was almost comparable to the baseline (Fig. 4).

Table 6 shows the summary statistics of PD parameters and their inter-group differences. The two-sided 90% CI for inter-group differences demonstrated a lower Ae_0–48_ in each hepatic impairment groups than in the normal hepatic function group. ∆EC_max_ and ∆AUEC_0–48_ were lower only in the severe hepatic impairment group. FEUA_0–24_/FEUA_−24–0_ was higher only in the moderate hepatic impairment group compared to the normal hepatic function group. No significant differences in the other PD parameters were observed between the normal hepatic function group and the hepatic impairment groups.

**Table 6:** Summary statistics of pharmacodynamic parameters and differences between groups

Parameters (unit)	Normal hepatic function (*n* = 6)	Mild hepatic impairment (*n* = 6)		
	Mean ± SD	Mean ± SD	Mean difference between groups^a^	
			Point estimate	Two-sided 90% CI
ΔEC_max_ (mg/dL)	− 3.52 ± 0.74	− 3.08 ± 0.37	0.43	− 0.36 to 1.23
ΔAUEC_0–48_ (mg h/dL)	− 135.01 ± 29.77	− 118.79 ± 21.12	16.22	− 16.67 to 49.10
Ae_0–48_ (mg)	1836.37 ± 357.99	1481.72 ± 381.54	− 354.65	− 632.52 to 76.78
FEUA_-24–0_ (%)	3.45 ± 0.59	2.89 ± 0.79	− 0.56	− 2.02 to 0.90
FEUA_0–24_ (%)	16.19 ± 2.93	14.51 ± 2.10	− 1.68	− 5.39 to 2.02
FEUA_24–48_ (%)	6.53 ± 1.73	5.62 ± 1.00	− 0.92	− 3.25 to 1.42
FEUA_0–24_ /FEUA_-24–0_	4.710 ± 0.526	5.257 ± 1.172	0.547	− 0.891 to 1.985
FEUA_24-48_/FEUA_-24–0_	1.886 ± 0.323	2.050 ± 0.556	0.164	− 0.376 to 0.705

Parameters (unit)	Moderate hepatic impairment (*n* = 9)			Severe hepatic impairment (*n* = 3)		
	Mean ± SD	Mean difference between groups^a^		Mean ± SD	Mean difference between groups^a^	
		Point estimate	Two-sided 90% CI		Point estimate	Two-sided 90% CI
ΔEC_max_ (mg/dL)	− 3.66 ± 0.97	− 0.14	− 0.86 to 0.59	− 2.27 ± 0.93	1.25	0.28 to 2.22
ΔAUEC_0-48_ (mg·hr/dL)	− 143.48 ± 39.32	− 8.48	− 38.49 to 21.54	− 86.23 ± 37.31	48.78	8.50 to 89.05
Ae_0–48_ (mg)	1466.66 ± 152.10	− 369.71	− 623.37 to 116.06	1125.08 ± 42.72	− 711.29	− 1051.61 to 370.97
FEUA_−24–0_ (%)	2.29 ± 1.00	− 1.16	− 2.49 to 0.18	4.74 ± 3.88	1.29	− 0.50 to 3.08
FEUA_0–24_ (%)	13.12 ± 3.10	− 3.07	− 6.45 to 0.31	12.75 ± 8.22	− 3.44	− 7.98 to 1.09
FEUA_24–48_ (%)	5.26 ± 2.94	− 1.28	− 3.41 to 0.86	6.77 ± 3.24	0.23	− 2.63 to 3.10
FEUA_0–24_ /FEUA_−24–0_	6.377 ± 1.867	1.667	0.354− 2.980	3.048 ± 1.669	− 1.662	− 3.423 to 0.099
FEUA_24–48_ /FEUA_−24–0_	2.202 ± 0.546	0.317	− 0.177 to 0.810	1.733 ± 0.849	− 0.153	− 0.815 to 0.509

^a^The mean difference was calculated using the following formula:

Mean difference = (mean in the target group) − (mean in the normal hepatic function group)

*Ae*_*0–48*_ amount of uric acid excreted in urine from time 0 to 48 h, *∆AUEC*_*0–48*_ delta area under the serum uric acid concentration–time curve from time 0 to 48 h, *CI* confidence interval, *∆EC*_*max*_ delta maximum effective concentration, *FEUA*_*t1–t2*_ fractional excretion of uric acid from time t1 to t2

### Safety

A total of two AEs were reported in the moderate impairment group: one event of renal impairment in one subject and one event of abdominal pain in one subject. Both events were considered to be ADRs. Renal impairment was moderate in severity and abdominal pain was mild in severity. All AEs were considered to have resolved at follow-up examination. Overall, dotinurad was safe without any concerns related to laboratory values or vital signs.

## Discussion

This study evaluated the PK, PD, and safety of a single oral dose of dotinurad 4 mg in subjects with hepatic impairment relative to those with normal hepatic function.

Subjects with hepatic impairment were divided into three groups based on severity. The Child–Pugh classification, recommended by the Food and Drug Administration Guidance for Industry Pharmacokinetics in Patients with Impaired Hepatic Function [12], was used in classifying the severity of hepatic impairment. Since the Child–Pugh classification is a scoring system for determining the severity of cirrhosis, patients with cirrhosis were enrolled in hepatic impairment groups in the study.

In Japan, there have been few reports of clinical pharmacology studies in subjects with severe hepatic impairment. It is anticipated that enrolling subjects with severe hepatic impairment is difficult, so we continued the enrollment of such subjects until the end of study treatment in other study groups. As a result, the study enrolled six subjects with normal hepatic function, six with mild hepatic impairment, nine with moderate hepatic impairment, and three with severe hepatic impairment.

Time courses of plasma dotinurad levels in the hepatic impairment groups are similar to those in normal hepatic function. *C*_max_ of moderate and severe hepatic impairment groups are lower than that of normal hepatic function group. No significant differences were noted in the other PK parameters between subjects with hepatic impairment and those with normal hepatic function. Subjects with moderate and severe hepatic impairment had a higher unbound fraction in plasma than those with normal hepatic function. An in vitro study revealed albumin as the primary binding protein for dotinurad (data not shown). In light of the negative correlation between the unbound fraction in plasma and serum albumin levels, a higher unbound fraction in plasma, in subjects with moderate and severe hepatic impairment, could be explained by reduced production of albumin due to impaired hepatic function. This could also relate to a lower *C*_max_ in subjects with moderate and severe hepatic impairment. Specifically, a reduction in *C*_max_ may have resulted from augmented clearance and volume of distribution due to an increased proportion of unbound drug. Other possible factor was decreased absorption due to intestinal edema associated with hepatic disorders. A clinical pharmacology study of dotinurad in elderly men and women identified body weight as a factor for PK variation [NCT#02344875]. We also suspected that body weight could cause lower *C*_max_ in the present study, and adjusted plasma dotinurad levels and PK parameters for body weight and then compared them between subjects with normal hepatic function and those with hepatic impairment. The adjusted values in subjects with severe hepatic impairment were almost comparable to those in subjects with normal hepatic function (Table 3). This finding suggested that differences in body weight influence on lower *C*_max_ in subjects with severe hepatic impairment.

All groups had a similar fe for the glucuronate conjugate, a urinary metabolite of dotinurad. Subjects with moderate hepatic impairment had a lower fe for the sulfate conjugate than those with normal hepatic function, whereas the fe in subjects with mild and severe hepatic impairment was comparable to that in those with normal hepatic function. No significant inter-group differences in CL_tot_/*F* of dotinurad were observed, indicating little involvement of sulfate conjugation. These findings suggest that hepatic impairment may not meaningfully affect the metabolism of dotinurad. In general progression of hepatic disorders reportedly lowers oxidative metabolic activity but not glucuronidation activity [13]; our results agree with this report. Based on the finding that the excretion rate of the primary metabolite glucuronate conjugate was similar in all groups in the study, there seemed to be no meaningful differences in the absorption of dotinurad between subjects with hepatic impairment and those with normal hepatic function.

Regarding pharmacodynamics of dotinurad, serum uric acid levels varied at baseline between the groups but followed a similar postdose time course in all groups. Only severe hepatic impairment group had lower ∆EC_max_ and ∆AUEC_0–48_ than those with normal hepatic function. This was probably due to lower baseline serum uric acid levels in the severe hepatic impairment group than in the other groups. With regard to urinary uric acid excretion, hepatic impairment groups had lower Ae_0–48_ than normal hepatic function group, whereas no significant difference in FEUA was observed.

No clear differences in ∆EC_max_ and ∆AUEC_0–48_ were noted between the mild and moderate hepatic impairment groups and the normal hepatic function group, and lower Ae_0–48_ in these groups did not appear to affect the reduction in serum uric acid concentration. Lower Ae_0–48_ in subjects with severe hepatic impairment was probably due to lower baseline serum uric acid levels in this group than in other groups. No significant inter-group differences in the other PD parameters were observed. Overall, no clear PD differences were observed between groups.

In summary, hepatic impairment appeared to be associated with reduced *C*_max_ and increased unbound fraction in plasma but did not meaningfully influence on the other PK parameters or PD parameters. These findings indicate that hepatic impairment has no clinically relevant influence on the PK of dotinurad and does not attenuate efficacy.

Two subjects with moderate hepatic impairment experienced two AEs, for which a causal relationship with dotinurad could not be ruled out. One of the two events, renal impairment, was considered to be an AE based on an elevation in serum creatinine levels from 1.16 mg/dL (baseline) to 1.61 mg/dL (day 2). Following fluid replacement from day 2 to 3, the serum creatinine level dropped to 0.91 mg/dL at follow-up examination (day 8) and the event was considered to have resolved. There were no noteworthy findings regarding other safety measures. Taken together, a single oral dose of dotinurad 4 mg was safe in subjects with hepatic impairment without any major concerns.

In conclusion, the study revealed no clinically relevant influence of hepatic impairment on the PK, PD, or safety of dotinurad. These findings indicate possibility that dotinurad can be used without dose adjustment in patients with hepatic impairment.

## Electronic supplementary material

Below is the link to the electronic supplementary material.

Supplementary file1 (DOCX 50 kb)

Supplementary file2 (DOCX 45 kb)
