# Supplementary material for: Dotinurad: a clinical pharmacokinetic study of a novel, selective urate reabsorption inhibitor in subjects with hepatic impairment
Source: Clin Exp Nephrol. 2019 Nov 23;24(Suppl 1):25–35. doi: 10.1007/s10157-019-01816-4 (PMC7066095; doi:10.1007/s10157-019-01816-4)
Supplement: Supplementary file 2 — Supplementary file2 (DOCX 45 kb) [file 10157_2019_1816_MOESM2_ESM.docx]

**Supplement 2** Summary statistics of plasma dotinurad levels (adjusted for body weight)

| Time postdose (hr) | Plasma dotinurad levels adjusted for body weight ^a)^ (ng/mL, Mean ± SD) | | | | | | | | | | | |
| --- | --- | --- | --- | --- | --- | --- | --- | --- | --- | --- | --- | --- |
|  | Normal hepatic function (n = 6) | | | Mild hepatic impairment (n = 6) | | | Moderate hepatic impairment (n = 9) | | | Severe hepatic impairment (n = 3) | | |
| Baseline | 0.00 | ± | 0.00 | 0.00 | ± | 0.00 | 0.00 | ± | 0.00 | 0.00 | ± | 0.00 |
| 0.5 | 128.02 | ± | 56.79 | 133.21 | ± | 129.74 | 171.14 | ± | 111.15 | 217.09 | ± | 99.25 |
| 1 | 227.59 | ± | 75.17 | 210.30 | ± | 142.88 | 230.25 | ± | 97.34 | 295.57 | ± | 62.71 |
| 2 | 282.15 | ± | 96.40 | 282.41 | ± | 112.17 | 267.29 | ± | 85.02 | 295.64 | ± | 69.02 |
| 3 | 312.31 | ± | 41.46 | 279.72 | ± | 82.74 | 260.96 | ± | 66.06 | 275.80 | ± | 68.29 |
| 4 | 309.06 | ± | 39.11 | 273.14 | ± | 45.79 | 251.85 | ± | 67.60 | 260.37 | ± | 63.39 |
| 6 | 235.20 | ± | 38.54 | 205.45 | ± | 35.27 | 190.52 | ± | 49.87 | 203.07 | ± | 57.29 |
| 8 | 197.24 | ± | 32.12 | 179.41 | ± | 28.61 | 161.36 | ± | 46.65 | 163.25 | ± | 50.66 |
| 12 | 134.88 | ± | 22.84 | 135.07 | ± | 26.80 | 123.60 | ± | 35.04 | 127.84 | ± | 48.12 |
| 24 | 64.86 | ± | 5.37 | 65.55 | ± | 24.07 | 66.14 | ± | 31.52 | 62.02 | ± | 31.39 |
| 36 | 26.67 | ± | 2.97 | 26.51 | ± | 13.55 | 26.21 | ± | 16.36 | 23.93 | ± | 16.23 |
| 48 | 14.33 | ± | 1.54 | 14.43 | ± | 9.22 | 14.90 | ± | 10.72 | 12.21 | ± | 10.32 |

a) Plasma dotinurad level adjusted for body weight = drug concentration × baseline body weight / 60

SD, standard deviation
